# Optimal Bell’s Palsy Treatment: Steroids, Antivirals, and a Timely and Personalized Approach

**DOI:** 10.3390/jcm13010051

**Published:** 2023-12-21

**Authors:** Hwa Sung Rim, Jae Yong Byun, Sang Hoon Kim, Seung Geun Yeo

**Affiliations:** Department of Otorhinolaryngology—Head and Neck Surgery, College of Medicine, Kyung Hee University Medical Center, Seoul 02447, Republic of Korea; marslover@naver.com (H.S.R.); otorhino512@naver.com (J.Y.B.); hoon0700@naver.com (S.H.K.)

**Keywords:** Bell’s palsy, steroids, antivirals

## Abstract

Importance: The optimal treatment approach for patients with Bell’s palsy, a condition characterized by acute facial nerve palsy, remains unclear. The present study was designed to provide insights into the most effective treatment strategies, whether steroids alone or steroids plus antiviral agents, as well as the optimal timing of treatment initiation. Objective: To investigate the impact of treatment modalities and timing on the recovery rates of Bell’s palsy patients and to assess the roles of individual factors. Design, Setting, and Participants: This retrospective analysis included 1504 patients with Bell’s palsy who visited Kyung Hee University Hospital. Patients were divided based on the treatment modality (steroid monotherapy vs. combined steroid and antiviral therapy) and the timing of treatment initiation (≤72 vs. >72 h). Main Outcomes and Measures: The primary outcome was the recovery rate, as assessed by the House–Brackmann (HB) grade. Secondary outcomes included factors such as age, electroneurography (ENoG) and electromyography (EMG) results, and comorbid conditions. Results: A combined comparison of patients treated with steroids plus antivirals and steroids alone, stratified by treatment start time, showed that recovery rates were highest in patients who received steroid monotherapy initiated within 72 h (OR 2.36; *p* < 0.05). Patients with severe Bell’s palsy tended to benefit more from combined therapy when treatment was initiated within 72 h. The recovery rate was higher in patients who received steroid monotherapy than combined therapy (86.32% vs. 79.25%, *p* < 0.05). Initiating treatment beyond 72 h was associated with a higher recovery rate than starting treatment within 72 h (85.69% vs. 76.92%, *p* < 0.05). An evaluation of the factors affecting recovery showed that patients aged 20 to 39 years had a higher recovery rate than other age groups (OR 1.47; *p* < 0.05). Fairly predictive EMG results were associated with significantly higher recovery rates (OR 3.52; *p* < 0.05). Conclusions: These findings underscore the importance of individualized treatment approaches in Bell’s palsy management. Steroid monotherapy remains effective, although combined treatment may have potential advantages, especially in patients with more severe disease. The best treatment results were achieved when steroid treatment was administered within 72 h. Our results suggest that there may be more flexibility in the application of the 72 h treatment period if we consider the time of treatment initiation alone, but this should take into account patient behavior patterns and the limitations of retrospective analysis. Further research is warranted to validate these findings and refine treatment recommendations for patients with Bell’s palsy.

## 1. Introduction

Bell’s palsy is an acute onset of facial nerve paralysis with an unknown cause, affecting approximately 11–40 per 100,000 people [1,2]. It can significantly impact patients’ social activities and occurs roughly equally in men and women [3]. While it was once thought to be most common in individuals aged 30 to 45, recent research indicates a second peak of occurrence in those over 70 [4,5,6,7,8,9]. Pregnant women and individuals with conditions such as diabetes, influenza, or respiratory ailments are more prone to developing Bell’s palsy [10]. The precise etiology remains elusive, with proposed causes including viral infections, vascular problems, autoimmune inflammation, and hereditary anatomical anomalies [11,12,13,14,15]. Herpes simplex virus (HSV) has been implicated as a potential culprit, as it has been detected in the geniculate ganglia and endoneurial fluid of Bell’s palsy patients, suggesting a viral link [16,17,18,19,20].

Treatment guidelines have evolved over time. The 2013 guidelines recommended steroid monotherapy, with antiviral treatment alone not recommended [21]. However, subsequent studies have presented mixed results. Some indicated that combining steroids and antiviral agents yielded better recovery rates [22,23], while others found limited benefit in adding antivirals [24]. Long-term complications like crocodile tears and synkinesis were reported to be less common in patients receiving combination therapy [24]. A randomized trial even showed a higher complete recovery rate in patients treated with steroids and famciclovir compared to steroid monotherapy, suggesting the usefulness of antiviral drugs and supporting combination therapy for complete paralysis cases [25].

Overall, the effectiveness of combining steroids and antiviral agents in Bell’s palsy remains uncertain, leading to contradictory findings in previous research. This present study aimed to investigate the effectiveness of different treatment approaches, including steroids alone or in combination with antiviral agents, and the impact of treatment initiation timing in Bell’s palsy patients. The goal was to provide clarity on the most effective treatment strategies for this condition.

## 2. Methods

**Figure 1:**
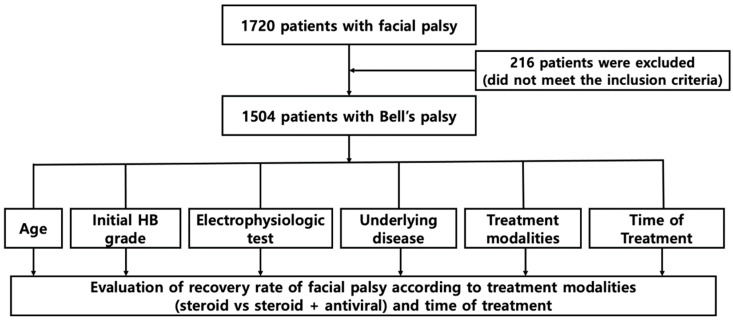
Flow diagram of the study design. Abbreviations: HB grade, House–Brackmann grade.

A retrospective analysis was conducted on 1708 patients diagnosed and hospitalized with Bell’s palsy at Kyung Hee University Hospital from January 1986 to May 2023. Exclusion criteria included congenital malformations, trauma, infections, otitis media, tumors, metabolic diseases, systemic diseases, autoimmune diseases, age below 20 years, unclear timing of Bell’s palsy onset, insufficient medical records, receiving only supportive management, and loss to follow-up. The patients were stratified based on gender, age at initial visit, initial House–Brackmann (HB) grade, electroneurography (ENoG) results, electromyography (EMG) results, presence or absence of diabetes and hypertension, treatment method, treatment start time, and recovery rate (Figure 1).

Patients in this study underwent facial nerve electroneurography (ENoG) 4 days after the onset of facial palsy symptoms, and all patients underwent electromyography (EMG) 2 weeks after the onset of symptoms. ENoG measurements were reported as a percentage, calculated as the amplitude on the affected side divided by the maximal amplitude on the normal (unaffected) side. A “poor” ENoG result was defined as a >90% loss of amplitude, indicating severe nerve damage. Conversely, a “good” outcome was defined as a ≤90% loss of amplitude, suggesting less severe nerve impairment [26].

Factors determined by a physical medicine and rehabilitation physician were also evaluated. These factors included the presence or absence of the blink reflex and the results of needle EMG tests on specific facial muscles, including the frontalis, orbicularis oculi, nasalis, and orbicularis oris muscles. The absence of pathological spontaneous activity (such as positive sharp waves or fibrillation potentials) on needle EMG tests was regarded as predictive of favorable outcomes, suggesting that the nerves and muscles were functioning reasonably well. In contrast, the presence of abnormal spontaneous activity or the absence of volitional (intentional) muscle activity was defined as predictive of poor outcomes, indicating more severe nerve and muscle dysfunction.

All patients with Bell’s palsy were treated with oral steroids, either alone or in combination with antiviral agents. The dosage for adults was 80 mg/day of oral steroids for the first four days, followed by a tapering regimen. Low-body-weight adults were administered 1 mg/kg oral steroids for the first four days, followed by dose reductions over the next seven days. When required, patients were also administered acyclovir or famciclovir. The degree of facial paralysis was evaluated using the HB grade, and patients were followed up for at least six months. Recovery to HB grades I and II was defined as satisfactory. The study protocol was approved by the Institutional Review Board of Kyung Hee University Hospital (IRB No 2019-07-065), with the requirement for informed consent waived due to the retrospective nature of the study.

### Statistical Analysis

Categorical variables, including gender, were reported as frequencies and percentages and compared by chi-square tests, whereas continuous variables, including age, were reported as means ± standard deviations (SDs) and compared by *t*-tests. Factors associated with final recovery from facial nerve palsy were analyzed by univariate and multivariate logistic regression analyses, with results reported as odds ratios (ORs) and 95% confidence intervals (CIs). All statistical analyses were performed using SPSS version 20.0 (IBM Corp., Armonk, NY, USA), with *p*-values < 0.05 considered statistically significant.

## 3. Results

### 3.1. Characteristics by the Treatment Modalities

In this study on the recovery of patients diagnosed with Bell’s palsy, 1504 individuals were evaluated. The group comprised 703 (46.7%) men and 801 (53.3%) women, with 82.22% of men and 82.90% of women showing favorable recovery. No significant difference in recovery rates was observed between men and women (*p* > 0.05). This study also compared the recovery rates of patients treated with steroids alone (709 patients, 47.1%) to those treated with a combination of steroids and antiviral agents (795 patients, 52.9%).

Analysis showed no significant differences in age distribution between the two treatment groups (*p* > 0.05). Regarding initial Bell’s palsy severity, both treatment groups were similar (*p* > 0.05). However, significantly more patients treated with steroids alone (86.32%) achieved favorable results (HB grade I or II) compared to those treated with steroids plus antiviral agents (79.25%) (*p* < 0.05). Patients with diabetes were more prevalent in the steroids plus antiviral agents group (15.85% vs. 13.64%, *p* < 0.05), as were those with controlled hypertension (32.83% vs. 43.30%, *p* < 0.05). Electroneuronography (ENoG) and electromyography (EMG) results at days 4 and 14 post onset, respectively, showed no significant difference between the two treatment groups for ENoG (*p* > 0.05), but a significant difference for EMG, with the steroids-alone group having fairer predictive values (80.54% vs. 50.94%, *p* < 0.05) (Table 1).

### 3.2. Characteristics by the Onset of Treatment

In this study of 1504 patients diagnosed with Bell’s palsy, 533 (35.4%) began treatment within 72 h, and 971 (64.6%) began treatment after 72 h. No significant differences in age distribution were found between the two groups (*p* > 0.05). The analysis showed no significant differences in the severity of Bell’s palsy between patients treated with steroids alone and those treated with steroids plus antiviral agents (*p* > 0.05). In terms of timing, 76.92% of patients who began treatment within 72 h had slight to moderate Bell’s palsy, compared to 85.68% of those who began treatment after 72 h, with no significant difference between these groups (*p* > 0.05). However, the difference in favorable outcomes between those who began treatment within 72 h (76.92%) and those who began treatment after 72 h (85.69%) was statistically significant (*p* < 0.05). Diabetes prevalence was similar in both groups (*p* > 0.05), but controlled hypertension was more prevalent in the >72 h group (*p* < 0.05). ENoG and EMG results were significantly different between the two groups, with better outcomes for patients who began treatment after 72 h (*p* < 0.05) (Table 1).

### 3.3. Comparison of Factors Affecting the Rate of Recovery from Bell’s Palsy

The highest recovery rate was seen in patients treated with steroids alone within 72 h of onset (OR 2.36; *p* < 0.05). There was no significant difference in recovery rates between genders or between patients with electroneuronography (ENoG) results < 10% and ≥ 10%. However, patients with fairly predictive electromyography (EMG) results had significantly higher recovery rates than those with non-fairly predictive results (OR 3.52; *p* < 0.05). Individuals were categorized into three groups—20–39 years, 40–65 years, and >65 years—and their recovery rates were compared. The highest recovery rate was observed in the group aged 20–39 years (OR 1.47; *p* < 0.05) (Table 2).

### 3.4. Recovery Rate According to Treatment Modality and Onset Based on the Initial Severity of Bell’s Palsy

In this study of patients with Bell’s palsy, the recovery rate was significantly higher for those with slight to moderate symptoms who were treated with steroids alone compared to steroids plus antiviral agents (OR 2.43; *p* < 0.05). However, for patients with severe symptoms, the recovery rate was higher with the combination of steroids and antiviral agents, although this difference was not statistically significant (*p* > 0.05). Additionally, patients with mild to moderate symptoms who started treatment after 72 h had a significantly higher recovery rate than those who started within 72 h (OR 2.44; *p* < 0.05), while the recovery rate for patients with severe symptoms was higher when treatment was initiated within 72 h, though this difference was not statistically significant (*p* > 0.05). The interaction between the initial severity of symptoms and recovery rate was statistically significant (*p*-value for interaction < 0.05) (Table 3).

## 4. Discussion

Although various approaches are used to treat Bell’s palsy, a definitive cure remains elusive. The analysis of pathological and microsurgical studies has provided important insights into the disease process. Notably, the acute phase of Bell’s palsy is characterized by pronounced edema or swelling inside the facial nerve canal [27,28]. Due to their anti-inflammatory properties, steroids play a crucial role in Bell’s palsy management. These drugs have the potential to reduce nerve swelling within the facial nerve canal. This is of paramount importance, because the reduction in swelling lowers pressure on the blood vessels, thereby improving blood circulation to the affected nerves. Despite studies showing the effectiveness of steroids in the management of Bell’s palsy [29,30,31], its etiology is of great interest. Bell’s palsy has been linked to viral infections, particularly HSV infection. HSV has been detected in the endoneurial fluid of many patients with Bell’s palsy, suggesting that neuroinflammation, a key characteristic of Bell’s palsy, may be secondary to HSV infection [17]. These findings suggested that combinations of steroids and antiviral drugs may be effective in the treatment of Bell’s palsy, with steroids suppressing inflammatory responses and nerve swelling and antiviral drugs targeting the potential viral etiology. This two-pronged approach addresses both the immediate symptoms and the possible viral causes of Bell’s palsy. Although these combinations have shown promising results, more rigorous and extensive research is required. Ensuring optimal doses, understanding potential side effects, and determining the exact duration of treatment will be crucial.

Our research group investigated the efficacy of various treatment approaches to Bell’s palsy, with a particular focus on combinations of steroids and antiviral drugs. The results of these prospective studies have contributed significantly to our understanding of the management of Bell’s palsy. For example, a previous prospective study provided early evidence that the combination of steroids and acyclovir was more effective than steroids alone in patients with severe Bell’s palsy [32]. These combinations may have distinct advantages, especially in patients with severe-to-complete Bell’s palsy [25], suggesting the need for tailored treatment strategies based on the severity of the condition. In addition, combinations of steroids and antiviral agents were found to be more effective for patients with severe Bell’s palsy who did not than did have comorbid conditions, such as hypertension or diabetes [33], indicating the importance of considering patients’ overall health in treatment decisions. In addition, combinations of steroids and antiviral agents were found to be effective in patients aged >40 years with severe Bell’s palsy and hypertension or diabetes [34], suggesting that this treatment approach could benefit older patients with additional health concerns.

Despite evidence showing the effectiveness of combination treatment, the therapeutic benefits of antiviral drugs in the treatment of Bell’s palsy remain unclear. For example, a 2019 Cochrane review of 14 randomized controlled trials involving 766 patients with varying degrees of Bell’s palsy found that the rate of incomplete recovery did not differ in comparing combinations of steroids and antiviral agents with steroid monotherapy [24]. However, this Cochrane review suggested that combination therapy may have benefits in preventing long-term complications associated with Bell’s palsy. This underscores the complexity of Bell’s palsy as a condition and the need for further research to clarify its optimal treatment strategies. Well-designed randomized controlled trials with appropriate statistical power are needed to definitively determine the efficacy of antiviral drugs in Bell’s palsy treatment. These trials should consider factors such as patient demographics, disease severity, and comorbid conditions to tailor treatment recommendations more precisely.

The present retrospective analysis was designed to provide further insights into treatment options by comparing the efficacy of steroid monotherapy with combined steroid and antiviral therapy in a cohort of 1504 patients with Bell’s palsy. The results of this study may inform clinical decision-making in the management of this condition. One of the key findings of this analysis was that the recovery rate was significantly higher in patients administered steroid monotherapy within 72 h than in the other three groups. In addition, multiple regression analysis found that the recovery rate was higher in patients aged 20 to 39 years than in other age groups when adjusted for the initial severity of disease. Another critical aspect of this study was the use of EMG to assess prognosis. The results showed that patients with a positive EMG prognosis had a significantly higher recovery rate, suggesting that EMG may be valuable in predicting treatment outcomes and guiding clinical decisions in the management of Bell’s palsy.

The subgroup analysis conducted in this study provided further information on the differential responses to treatment of Bell’s palsy patients based on their initial severity of disease. This finding emphasizes the importance of considering individualized treatment approaches based on factors other than age. For example, the choice between steroid monotherapy and combined steroid and antiviral therapy may depend on the initial severity of Bell’s palsy. Steroid monotherapy resulted in a significantly higher recovery rate than combined therapy in patients presenting with mild to moderate Bell’s palsy. Thus, steroid monotherapy is effective as a first-line treatment for patients with less severe disease, in which inflammation may play a more dominant role than viral infection. Interestingly, this analysis revealed an intriguing pattern in patients who started treatment > 72 h after symptom onset. These patients exhibited enhanced recovery rates, suggesting that delaying the start of treatment may yield more favorable outcomes, particularly in patients with mild to moderate disease. This finding challenges the conventional wisdom that early intervention is crucial in the management of all patients with Bell’s palsy, and indicates that patients should not be discouraged from seeking treatment beyond the initial 72 h window, especially if their condition is less than severe.

Conversely, an opposite pattern was observed in patients presenting with severe Bell’s palsy. Although not statistically significant, recovery rates were higher in patients treated with combined steroid and antiviral therapy, particularly when treatment was initiated within 72 h. This finding indicates that viral infection may play a more prominent role in patients with severe Bell’s palsy, with early initiation of combination therapy being more effective. Although these findings suggest that treatment strategies should be tailored based on the initial severity of Bell’s palsy, differences were not always statistically significant. Additional studies with larger sample sizes are therefore needed to confirm these trends.

Subgroup analysis revealed the complexity of Bell’s palsy, suggesting that the optimal treatment approach may vary based on the initial severity of the condition. Steroid monotherapy appears to be more beneficial for patients with mild to moderate disease, even when treatment initiation is delayed, whereas combination therapy may show promise for patients with severe Bell’s palsy, particularly when initiated early. These nuanced findings emphasize the importance of individualized treatment of Bell’s palsy, with additional studies required to confirm their clinical significance. The optimal treatment for Bell’s palsy remains unclear, as studies have reported conflicting results. These differences underscore the need for comprehensive analyses of previous results, as well as further investigations. For example, several studies have observed higher recovery rates in patients administered combined steroid and antiviral therapy than in those administered steroid monotherapy [32,35,36,37]. These findings suggest that simultaneously addressing both the inflammatory component of Bell’s palsy with steroids and the potential viral etiology with antiviral drugs may contribute to more favorable outcomes. In contrast, other studies have reported no significant difference in recovery rates between the two treatment approaches [22,23,38,39,40], indicating that the addition of antiviral drugs did not confer a substantial advantage over steroid monotherapy. Combined steroid and antiviral therapy, however, was found to result in higher recovery rates in a subset of patients with severe or severe to complete Bell’s palsy [25,41,42], indicating that the choice of treatment approaches may be influenced by the severity of Bell’s palsy, with more aggressive combination therapy warranted in patients with more severe disease. These differences in research findings highlight the multifaceted nature of Bell’s palsy and the potential variability in responses to treatment among different patient populations. Treatment decisions should therefore be based on individual patient profiles, including disease severity. Large-scale, well-designed randomized controlled trials are also needed to provide more definitive guidance on the most effective treatment strategies for Bell’s palsy.

The timing of treatment initiation in Bell’s palsy remains unclear, although guidelines of the American Academy of Otolaryngology-Head and Neck Surgery Foundation (AAO-HNSF) have recommended that combined treatment be initiated within 72 h of symptom onset [21]. In contrast, the results of the present study found that initiating treatment at later times was associated with a more favorable recovery rate. Mild-to-moderate-severity Bell’s palsy, however, is characterized by a high spontaneous recovery rate, suggesting that factors other than the timing of treatment may affect recovery outcomes. Although initiating treatment within 72 h was found to yield higher recovery rates in patients with severe Bell’s palsy, later treatment initiation may result in higher recovery rates in patients with mild to moderate Bell’s palsy.

The present study had several limitations, primarily its retrospective design. Biases related to patient behavior and early medical intervention, particularly in patients with severe Bell’s palsy, may have influenced recovery rates. Patients with more severe symptoms might have been more inclined to seek immediate medical attention, potentially leading to a skewed interpretation of study outcomes. Therefore, additional well-designed studies are necessary to not only clarify the timing of treatment for Bell’s palsy but also to identify the specific factors that contribute to recovery outcomes, all while considering the condition’s inherent tendency toward spontaneous recovery.

## 5. Conclusions

These findings suggest that the selection of treatment approach and timing in Bell’s palsy management should be informed by individual patient characteristics, including the severity of the condition. Favorable recovery outcomes were associated with treatment initiation within 72 h and steroid monotherapy. However, combinations of steroids and antiviral agents may have potential benefits, especially in patients with severe Bell’s palsy. Treatment of Bell’s palsy should be based on individual patient characteristics.

## Figures and Tables

**Table 1:** Baseline characteristics by treatment modality and onset of treatment of Bell’s palsy.

		Treatment Modalities, n (%)			Onset of Treatment, n (%)		
		Steroids Alone	Steroids Plus Antiviral Agents	*p*-Value †	≤72 h	>72 h	*p*-Value †
Sex	Males	322 (45.42%)	381 (47.92%)	0.3304	246 (46.15%)	457 (47.06%)	0.7348
	Females	387 (54.58%)	414 (52.08%)		287 (53.85%)	514 (52.94%)	
Age	20 ≤ age ≤ 39	184 (25.95%)	201 (25.28%)	0.8884	134 (25.14%)	251 (25.85%)	0.8844
	40 ≤ age ≤ 65	332 (46.83%)	369 (46.42%)		253 (47.47%)	448 (46.14%)	
	>65	193 (27.22%)	225 (28.30%)		146 (27.39%)	272 (28.01%)	
Initial state	Slight to moderate	565 (76.69%)	660 (83.02%)	0.0973	445 (83.49%)	780 (80.33%)	0.1315
	Severe	144 (20.31%)	135 (16.98%)		88 (16.51%)	191 (19.67%)	
DM	No	628 (88.58%)	669 (84.15%)	0.0129 *	454 (85.18%)	843 (86.82%)	0.3774
	Yes	81 (11.42%)	126 (15.85%)		79 (14.82%)	128 (13.18%)	
Controlled HTN	No	402 (56.70%)	534 (67.17%)	<0.0001 *	362 (67.92%)	574 (59.11%)	0.0008 *
	Yes	307 (43.30%)	261 (32.83%)		171 (32.08%)	397 (40.89%)	
Initial ENoG	≥10%	663 (93.51%)	754 (94.84%)	0.2698	487 (91.37%)	930 (95.78%)	0.0005 *
	<10%	46 (6.49%)	41 (5.16%)		46 (8.63%)	41 (4.22%)	
EMG	Poorly ^a^ predict	138 (19.46%)	390 (49.06%)	<0.0001 *	304 (57.04%)	224 (23.07%)	<0.0001 *
	Fairly ^b^ predict	571 (80.54%)	405 (50.94%)		229 (42.96%)	747 (6.93%)	

Abbreviations: DM, diabetes mellitus; HTN, hypertension, ENoG, electroneurography; EMG, electromyography. † Evaluated by chi-square tests. ^a^ Poorly predict is defined as presence of pathological spontaneous activity (such as positive sharp waves or fibrillation potentials). ^b^ Fairly predict is defined as absence of pathological spontaneous activity (such as positive sharp waves or fibrillation potentials). * Statistically significant. *p* < 0.05.

**Table 2:** Comparison of 6-month recovery from facial palsy according to variables in Bell’s palsy patients.

		Final Result			Favorable Recovery			
		Favorable Recovery ^a^	Unfavorable Recovery	*p*-Value †	OR	CI		*p*-Value ‡
Treatment modality and onset	≤72 h and steroids alone	118 (86.8%)	18 (13.2%)	<0.0001 *	2.36	1.34	4.19	0.0005 *
	≤72 h and steroids plus antiviral agents	292 (73.6%)	105 (26.4%)		1			
	>72 h and steroids alone	494 (86.2%)	79 (30.15%)		2.16	1.52	3.06	<0.0001 *
	>72 h and steroids plus antiviral agents	338 (84.9%)	60 (15.1%)		2.07	1.42	3.00	<0.0001 *
Sex	Males	578 (82.2%)	125 (17.8%)	0.7297	1			
	Females	664 (82.9%)	137 (17.1%)		1.05	0.8	1.38	0.7363
Age	20 ≤ age ≤ 39	328 (93.0%)	57 (7.0%)	0.09	1.47	1.01	2.14	0.0432 *
	40 ≤ age ≤ 65	582 (83.0%)	119 (17.0%)		1.22	0.89	1.67	0.2202
	>65	332 (79.4%)	86 (20.6%)		1			
Initial ENoG	≥10%	1169 (82.5%)	248 (17.5%)	0.7365	1			
	<10%	73 (83.9%)	14 (16.1%)		1.11	0.61	2.02	0.7375
EMG	Poorly predict	375 (71.0%)	153 (29.0%)	<0.0001 *	1			
	Fairly predict	867 (88.8%)	109 (11.2%)		3.52	2.66	4.68	<0.0001 *

Abbreviations: ENoG, electroneurography; EMG, electromyography; OR, odds ratio; CI, confidence interval. ^a^ Favorable recovery was defined as an HB grade I–II. † Evaluated by chi-square tests. ‡ Evaluated by multiple logistic regression. * Statistically significant. *p* < 0.05.

**Table 3:** Subanalysis of Bell’s palsy favorable recovery according to treatment modality and onset of treatment by Bell’s palsy initial state.

Initial State		Favorable Recovery ^a^								
		Slight to Moderate (HB Grade II–IV)				Severe (HB Grade V–VI)				*p*-Value of Interaction †
		OR	CI		*p*-Value †	OR	CI		*p*-Value †	
Treatment modality	Steroid monotherapy	2.43	1.70	3.48	<0.0001 *	0.82	0.49	1.37	0.4513	0.0008 *
	Combined steroid and antiviral therapy	1.00				1.00				
Onset of treatment	≤72 h	1.00				1.00				
	>72 h	2.44	1.75	3.39	<0.0001 *	0.99	0.57	1.73	0.9810	0.0086 *

Abbreviations: HB grade, House–Brackmann grade; OR, odds ratio; CI, confidence interval. ^a^ Favorable recovery was defined as an HB grade I–II. † Evaluated by multiple logistic regression. * Statistically significant. *p* < 0.05.

## Data Availability

The data presented in this study are available on request from the corresponding author. The data are not publicly available, as it is private clinical data.
